# Antibacterial Activity study of Musizin isolated from Rhamnus wightii Wight and Arn.

**DOI:** 10.6026/97320630014511

**Published:** 2018-12-21

**Authors:** William Raja Tharsius Raja, Stalin Antony, SaravanaKumar Pachaiyappan, Jackson Amalraj, Poorva Narasimhan, Balakrishna Keduki, Duraipandiyan Veeramuthu, Palani Perumal, Ignacimuthu Savarimuthu

**Affiliations:** 1 Division of Ethnopharmacology, Entomology Research Institute, Loyola College, Chennai; 2 2Division of Bioinformatics,Entomology Research Institute, Loyola College, Chennai; 3 Division of Microbiology, Entomology Research Institute, LoyolaCollege, Chennai - Chennai; 4 Department of Botany and Microbiology, Addiriyah Chair for Environmental Studies Collegeof Science, King Saud University, Riyadh 11451, Saudi Arabia; 5 Centre of Advanced Studies in Botany and Centre for Herbal Sciences,University of Madras, Guindy Campus, Chennai 600 025, India; 6 International Scientific Partnership Program, King Saud University, PostBox 2455, Riyadh 1011 11451, Kingdom of Saudi Arabia

**Keywords:** *Rhamnus wightii*, Musizin, antibacterial activity, target receptors, in silico analysis

## Abstract

The crude extracts and the compounds isolated from traditional medicinal plants are used to treat infectious diseases caused by bacteria, fungi, and viruses. An attempt has been made in the present investigation to evaluate the antibacterial activity of musizin isolated from Rhamnus wightii, (Family: Rhamnaceae) against Gram-positive (Bacillus cereus, Staphylococcus aureus, Streptococcus faecalis), and Gramnegative (*Escherichia coli*, * Klebsiella pneumonia*, and * Pseudomonas aeruginosa*) bacteria. The tested compound showed more pronounced antibacterial activity against the tested pathogens than the standard antibiotics like streptomycin and gentamycin with the lowest minimum inhibitory concentration (MIC). Molecular docking analysis was performed to study the effectiveness of musizin compared to the standard antibiotics; it showed a significant interaction with the target proteins such asalgR (*P. arginosa*), divIVA (*E. faecalis*), icaA (*S. aureus*), plcR(*B. cereus*), treC (*K. pneumonia*) and ftsl (*E. coli*) and found that musizin showed higher potential with least binding energy. It has also been found that musizin had better ADMET properties than the standard drugs. Thus,musizin acts as an inhibitor of bacterial growth for consideration as a drug to treat bacterial infections.

## Background

Antibiotics are one of the most important weapons in fighting against the bacterial infections and have greatly benefited the health-related quality of human life 1. However, the antibiotics which were used in ancient days have been found to be less effective against certain illnesses and even caused toxic reactions. In order to overcome these shortcomings, newer antibiotics need to be developed against which bacteria fail to develop resistance. Medications obtained from natural sources show a substantial role in the treatment of human illnesses. In many developing countries, traditional medicine has become an integral part of primary healthcare systems 2. It has been documented that the herbal plants play a vital role in traditional medicine and their curative potentials are tremendous 3. Between 1981 and 2002, the development of newer drugs (61%) from natural products was very successful, especially in the areas of infectious disease and cancer 4. Recent trends, however, show that the discovery rate of active novel chemical entities is declining and thus, natural medicine flourishes everywhere.

The crude extracts obtained from several plant species have shown antibacterial activity 5. Maheshwari et al. (1986) have done an appreciable level of work on ethno-medicinal plants in India 6. The antimicrobial activity of glycosides, a secondary metabolite, produced by plants has also been investigated using in vitro studies 7. Medications derived from natural sources assume a significant role in the prevention and treatment of human diseases. For instance, the utilization of bearberry (*Arctostaphyl osuva-ursi*) and cranberry (*Vaccinium macrocarpon*) juices to treat urinary tract contagions has been stated for in various manuals of phytotherapy, while species such as lemon balm (*Melissa officinalis*), garlic (*Allium sativum*) and tee tree (*Melaleucaal ternifolia*) have been reported to contain wide range antimicrobial agents 8. In the pursuit of identifying newer antibiotics, an effort has been made to find antimicrobial compound from a potential plant species, *Rhamnus wightii*.

*R. wightii* is a large shrub with brown bark, found in the hills of Peninsular India, up to an altitude of 2000m. In the Western Peninsula, the bark is much in repute on account of its tonic, astringent and deobstruent properties 9. Several active compounds such as cynodontin, chrysophanol, physcion, musizin, lupeol, sitosterol, 7-hydroxy-5 methoxyphthalide, emodin, and sitosterol glycoside have been isolated from the plant 9. The presence of compounds containing lactone ring such as 7-hydroxy- 5-methoxyphthalide and naphthalideglucoside has also been reported in this plant and more recently a new naphthalene glucoside lactone was isolated from the acetone extract of the stem bark of *Rhamnus wightii* 9.

Computational chemistry tools have become very important to ascertain the targets for different ligand molecules 10. It generates new knowledge that is useful in such fields as drug design and develops new software tools to create that knowledge. Experimental determination of efficacy and safety of antibiotics is a time and cost consuming procedure. Molecular docking analyses have proved efficient in ascertaining the functions of different ligand molecules and their biological functions 11-13. In the field of drug discovery, structural biology and computer-assisted drug design, molecular docking plays a major role and it has been widely used to identify the ligand-protein interactions; also it has been used to identifying the ligand binding sites on a protein in de novo drug design 14-15. Therefore, the main objective of the present investigation to perform ADMET studies with a phytochemical compound, musizin, isolated from *R. wightii* along with standard antibiotics such as streptomycin and gentamycin against bacterial pathogens. The molecular docking analysis was undertaken to identify the target proteins and the active binding sites in them from *Pseudomonas aerginosa*, *Enterococcus faecalis*, *Staphylococcus aureus*, *Bacillus cereus*, *Klebsiella pneumonia* and *Escherichia coli* and the results obtained have been presented.

## Methodology

### Plant material:

The plant material was collected from Naduvattam, Nilgiris District, Tamilnadu and was authenticated by Dr Pandikumar, taxonomist of the institute. A voucher specimen (No. RW-EA-02) has been deposited in the herbarium of the institute.

### Isolation of Musizin (1B):

Shade dried and coarsely powdered plant material (aerial part, leaves and stem, 3 kg) was extracted successively with hexane, chloroform,ethyl acetate and methanol in a Soxhlet apparatus. Extracts were filtered and concentrated in a rotary evaporator and finally dried in vacuum. The active ethyl acetate extract (yield 0.18%) was chromatographed over silica gel (s. d. fiNE - CHEM 100-200 mesh). The column was eluted with solvents of increasing polarity in the order hexane, chloroform and ethyl acetate their mixtures. Finally based upon TLC profiles,10 fractions were obtained. Fraction 2 eluted with hexane-chloroform 1:1 showed activity. Crystallization from hexane-chloroform mixture gave musizin (C13H12O3, MW: 216)as bright yellow crystals (mp 162-163°). The structure was confirmed by physical and spectroscopic data (UV, IR, 1H NMR, 13C NMR with DEPT and ESI-MS) as in our earlier publication 16.

### Determination of antibacterial activity and Minimum Inhibitory Concentration

### Test organisms:

The Gram-positive bacteria such as Staphylococcus aureusMTCC 96, Bacillus cereus MTCC 430, Enterococcus faecalisMTCC 439 and Gramnegative bacteria such as Klebsiella pneumonia MTCC 109, Pseudomonas aeruginosa MTCC 424 and Escherichia coli MTCC 726 were procured from the Institute of Microbial Technology (IMTECH), Chandigarh, India-160 036. The standard antibiotics purchased from Himedia.

### Inoculum preparation:

The bacterial pathogens were grown in Mueller Hinton broth (MHB; Hi-media, India) and obtained a standardized inoculum 17 which was used for antibacterial activity.

### Antibacterial activity testing:

The antimicrobial susceptibility testing with musizin and antibiotics were carried out using disc diffusion method 18 against six bacterial pathogens. The compound diffusion analysis was tested with different concentrations (1 and 2.5 mg/disc) of R. wightiicrude extracts. Simultaneously, the discs containing streptomycin (25μg/disc), gentamycin (50μg/disc) were used as standard antibiotics and 10% Dimethyl Sulfaoxide (DMSO) as negative control.The next day, the zone of inhibition (in mm) was recorded.

### MIC testing:

The MIC of musizin with different concentrations such as 1000, 500, 250, 125, 62.5, 31.25 and 15.62μg/mL was determined by two-fold dilution technique using a 96-well microtiter plate. Streptomycin and gentamycin were used as positive controls while DMSO as a solvent control and MHB as a negative control. The plates were incubated for 24h at 37°C. After incubation, 5μL of the tested broth was inoculated on plain Mueller Hinton agar plates to observe the viability of the test organism 19.

### Docking analysis:

### Ligand preparation:

The ligand musizin and the standard antibiotics, streptomycin and gentamycin were drawn in ChemDrawUltra version 12.0 assigned with proper 2D, 3D orientation without bond connection error. The energy of the molecules was minimized using PRODRG2-Server 20 and ADMET properties were predicted with Data Warrior software (www.openmolecules.org).

### Protein preparation and molecular docking:

Due to the unavailability of 3D structure of the target protein, swiss model server was used to develop a 3D model. The specific ID for each microorganism was allotted and sequences were retrieved from uniprotkb. They were IcaA from S.aureus(ID:A0A1D4ZB27), algR from P. aerginosa(ID:P26275), divIVA from E. Faecalis(ID:H7C713), plcR from B. cereus (ID:Q9XCQ6), treC from K. pneumoniae (ID:W9BQE5) and ftsl from E. coli(ID: P0AD68). The best-fit templates for these proteins sequence were recognized using BLAST analysis such as IcaA PDB-ID: 4HG6, algR-PDB-ID: 4CBV, divIVA�PDB-ID: 4XA6, plcR-PDB-ID: 2QFC, treC-PDB-ID: 5BRQ, ftsl-PDB-ID: 4BJP. After homology modeling, the best models were analyzedby PROCHECK-Ramachandran plot on SAVES server. The probable binding sites on the target receptors were searched using CASTp server 21. All the images and proteinligand interactions were visualized using PyMOL, (http://www.pymol.org).

The docking analysis was carried out using AutoDock Tools (ADT) v1.5.4 22 and AutoDock v4.2 programs with slight modification of the previous publication 13. The compound Musizin and the standard antibiotics streptomycin and gentamycin were docked to target modelled proteins with the molecule considered as a rigid body and the ligands being flexible.The hydrophobic effect of the ligand was retrieved by ProteinsPlus server (http://proteinsplus.zbh.uni-hamburg.de/).

### Statistical analysis:

Statistical results were calculated as mean±SD by SPSS 16.0. The significant differences were measured at P < 0.05.

## Results

### Identification of the compound using column chromatography:

**Figure 1:**
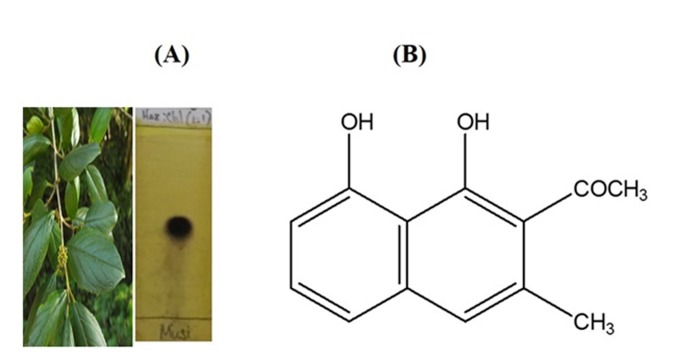
TLC profile and chemical structure of musizin from *R. wightii*

The Purification and Identification of Musizin (Figure 1) were reported previously 16 by Raja et al. 2018 (data available with authors). The ADMET properties of the ligand musizin and standard antibiotics, streptomycin and gentamycin, are presented in Table 1.

### Minimum inhibitory concentration (MIC):

### Antibacterial activity:

**Figure 2:**
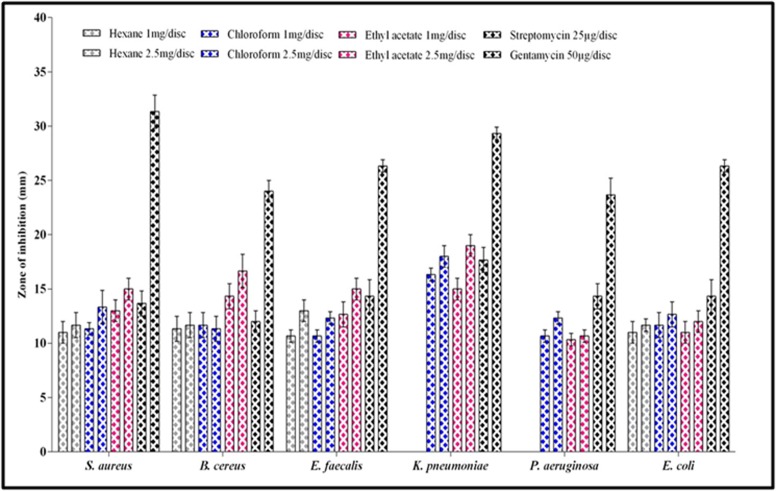
Antibacterial activity of different organic solvent extracts of *R. wightii* against human pathogenic bacteria

The antibacterial activity of R. wightii was tested against selected bacterial pathogens and their results are presented in Figure 2. The hexane and chloroform extracts showed lesser activity while methanol extract was found inactive against the tested bacteria. The ethyl acetate extract exhibited relatively higher and broad spectrum antibacterial activity with the zones of inhibition ranging from 10.66 ± 0.57 to 16.33±00 mm and 10.66 ± 0.57 to 19.00 ± 1.00 mm at 1.0 and 2.5mg/disc, respectively. The observed antibacterial activity of the extracts is highly comparable with the streptomycin (25µg/disc) and Gentamycin (50µg/disc) against *K. Pneumonia* with the zone of inhibition of 19.00 ± 1.00, followed by *B. cereus* (16.66 ± 1.52), *S. aureus*, *E. Faecalis* (15.00 ± 1.00), *E. coli* (12.00 ± 1.00) and *P. aeruginosa* (10.66 ± 0.57).

**Figure 3:**
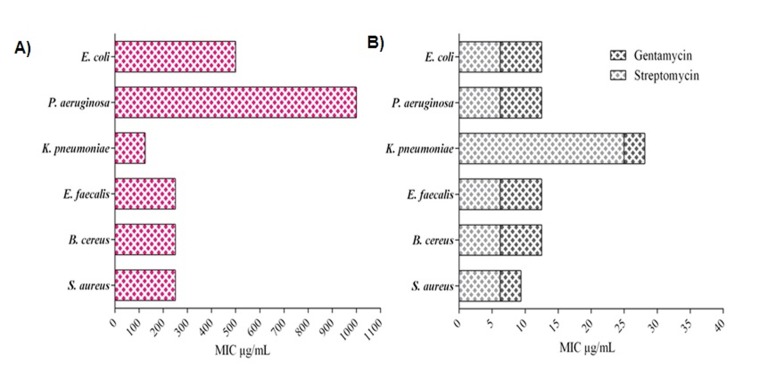
Determination of Minimum Inhibitory Concentration for purified Musizin (A) and standard antibiotics, gentamycin and streptomycin (B). *K. Pneumonia* showed highest sensitivity against Musizin at 120 µg per mL concentration.

The antibacterial activity shown by the extract obtained with ethyl acetate was further validated by bioassay-guided fractionation using MIC. The isolated compound musizin showed significant (P>0.05) minimum inhibitory concentration against K. pneumonia with MIC value of 125µg/mL followed by B. cereus S. aureus, E. faecalis(250 µg/mL each), E. coli (500 µg/mL) and P. aeruginosa(1000 µg/mL; Figure 3). The most antibiotic MIC values of musizin and the standard drugs are 120 µg/mL and 9 µg/mL against K. pneumoniaeand S. aureus respectively.

### Template identification and homology modelling:

**Figure 4:**
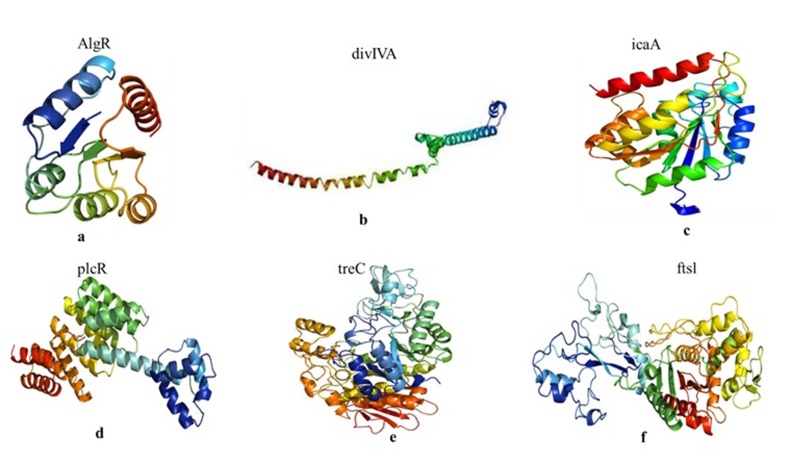
The figure represents the homology model of target proteins such asAlgR (a), divIVA (b), icaA (c), plcR (d), treC (e) and ftsl (f).

The target protein models created were examined by PROCHECK investigation using Ramachandran plot on SAVES server 23. The Ramachandran plot for algR protein demonstrated the amino acids deposits of 94.4% at a most favoured region, 4.7% in additional allowed regions and 0.9% in disallowed regions; there were no generously allowed regions. The Ramachandran plot for divIVA protein established the amino acids credits of 100 % at a most favoured region; all the other regions did not show the amino acids deposits.The Ramachandran plot for icaA protein established the amino acids gatherings of 85.0 % at a most favoured region, 12.2% in additional allowed regions, 2.3% in generously allowed regions; and 0.5 % in disallowed regions. The Ramachandran plot for plcR protein recognized the amino acids assemblies of 92.3% at the most favoured region, 6.3% in additional allowed regions, 1.1% in generously allowed regions; and 0.4% in disallowed regions. The Ramachandran plot for treC protein demonstrated the amino acids crowds of 89.7% at a most favoured region, 9.7% at additional allowed regions, 0.6% at generously allowed regions and there were no disallowed regions. The Ramachandran plot for ftslprotein confirmed the amino acids accumulations of 86.7% at a most favoured region, 11.5% in additional allowed regions, 1.2% in generously allowed regions; and 0.6% in disallowed regions.The modelled structures of all the target proteins were analysed in the RMSD range of 0.5 as shown in Figure 4.

### Molecular Docking Analysis:

The molecular docking analysis was performed to understand the possible binding interactions and atomistic events between the ligand, musizin and the receptor molecule on the bacterial membranes.The interaction of the compound musizin with the modelled algR active site is listed in Table 2. The hydrogen bonding interactions between musizin and active site residues of algR showed phenolic OH at C-1 and C-7 position interacted with GLU`9, phenolic OH at C-7 position interacted with LYS`102 and the acetyl carbonyl group at C-2 position interacted with ARG`15. The corresponding binding energy was observed as -4.51kcal/mol. and its inhibition constant value and ligand efficiency were 494.53 and 0.28, respectively.

The interactions of compound musizin with the modelled divIVA active site are listed in Table 2. The hydrogen bonding interactions between musizin and active site residues of divIVA showed phenolic OH at C-1 and C-7 positions interacted with PHE`13. The corresponding binding energy was observed as -6.09kcal/mol. and its inhibition constant value and ligand efficiency were 34.27 and 0.38, respectively.

The interactions of compound musizin with the modelled icaA active site are listed in Table 2. The hydrogen bonding interactions between musizin and active site residues of icaA showed the acetyl carbonyl at C-2 position, the phenolic OH at C-1 and C-7 positions interacted with SER`202, the phenolic OH at C-1 and C-7 position interacted with LYS 189. The corresponding binding energy was observed as -5.79kcal/mol. and its inhibition constant value and ligand efficiency were 57.28 and 0.36, respectively.

The interactions of compound musizin with the modelled plcR active site are listed in Table 2. The hydrogen bonding interactions between musizin and active site residues of plcR showed the phenolic OH at C-1 position interacted with LYS`87, the phenolic OH at C-7 position interacted with GLU1D. The corresponding binding energy was observed as-5.06kcal/mol. and its inhibition constant value and ligand efficiency were found to be 195.35 and 0.32 respectively.

The interactions of compound musizin with the modelled treC active site are listed in Table 2. The hydrogen bonding interactions between musizin and active site residues of treC showed the acetyl carbonyl at C-2 position that interacted with ASN` 63, a phenolic OH at C-1 and C-7 position interacted with GLN`168, C-7 phenolic OH interacted with HIS`105. The corresponding binding energy was observed as -5.63 kcal/mol. and its inhibition constant value and ligand efficiency were 74.94and 0.35, respectively.

The interactions of compound musizin with the modelled ftsl active site are listed in Table 2. The hydrogen bonding interactions between musizin and active site residues of ftsl showed phenolic OH at C-1 and acetyl carbonyl at C-2 position jointly interacted with ARG`71. The acetyl carbonyl at C-2 position interacted with SER`85, phenolic OH at C-1 and C-7 positions interacted with ASP`220, and a phenolic OH at C-1 position interacted with ILE`221. The corresponding binding energy observed was - 5.03kcal/mol. and its inhibition constant value and ligand efficiency were 204.48 and 0.31 respectively. The 3D and 2D images of the hydrophobic interaction of the compound musizin and control antibiotics like gentamycin (binding site interaction with active site RMSD value), streptomycin (binding site interaction with active site RMSD value), and docking results have been presented in Table 2.

## Discussion

The antibacterial activity of the extracts against K. pneumoniae is noteworthy because plant extracts and their compounds have previously been reported to be more active against Gram-positive bacteria than Gram-negative ones 24. Our results are also in agreement with the previous reports 25 of Carranza et al. (2015) who have shown a significant antibacterial activity of the leaf extract of Rhamnuscalifornica against two bacterial pathogens with the zones of inhibition ranging from 10.0 ± 2.1 to 14.0 ± 1.4 (mm). Similarly, the crude extract obtained from a Rhamnous member Ventilago madraspatana has been shown to have broad-spectrum antimicrobial activity against a panel of Gram positive, Gramnegative bacteria and Candida pathogens 26.

For the Minimum inhibitory concentration (MIC), the data obtained in the present study are in conjunction with an observation made previously 27 by Nishina *et al.* (1991) with a musizin isolated from Rumex japonicas that has shown antioxidant properties and R. aquaticus with antibacterial activity 28. To our knowledge, this is the first scientific report on the antibacterial activity from R. wightii. To support for the anti-bacterial activity, six types of modelled target proteins were subjected to molecular docking analysis and the results are very significant.Each protein has a different mechanism, so their mechanisms of action are also vary based on the inhibition of ligand molecules. AlgR is a transcriptional regulator of virulence factors in opportunistic human pathogen such as P. aeruginosa which regulates expression of a variety of genes including, type IV pilus function and alginate production indicating AlgR plays an important role in the regulation of gene expressions 29. divIVA homolog plays an essential role in maintaining proper cell division, and viability in *E. Faecalis* 30. It has been reported that DivIVA helps to position the oriC region of the chromosome at the cell pole in preparation for polar division 31.

icaA genes play a significant role in biofilm formation which mediates cell to cell adhesion (polysaccharide intracellular adhesion) in Staphylococcus species. Among the Ica genes, IcaA encodes N-acetyl glucosaminyl transferase, the enzyme involved in the synthesis of N-acetyl-glucosamine oligomers from UDP-Nacetylglucosamine which is extensively involved in intracellular signalling 32.plcR has been found to be a pleiotropic regulator of extracellular virulence in B. cereus. In addition, the non-hemolytic enterotoxin Nhe and the hemolysinsHbl, CerAB, CytK, CytK, Hbl and Nhe which are responsible for toxic infections with severe diarrheic and the phosphatidylinositol phospholipase-C PI-PLC. The genes encoding these proteins are under the control of theplcR 32.

treC encodes trehalose-specific phosphotransferase system enzyme IIB/IIC component in K. pneumonia which plays a crucial role during biofilm formation which contributes to the establishment, colonization and persistence of the bacterium in the gastrointestinal tract 33. ftsIis a trans-peptidase essential for the proteins involved in cell division which catalyzes the cross-linking of the peptidoglycan cell wall at the division septum. It is also involved in the biosynthesis of peptidoglycan layer of the bacterial cell wall. It has been reported that the inactivation of ftsI results in fatal imbalance of bacterial intracellular pressure which ultimately leads to cell division arrest 34.

The ADMET properties analyzed with the compound musizin have shown that this compound has antibacterial properties and could be developed as a potential antibacterial drug in future. It has been found that the standard antibiotics such as gentamycin and streptomycin have interacted with the same amino acids of the selected target proteins as like musizin however they act as protein synthesis inhibitors by binding to the 30s subunit of the bacterial ribosome. The results obtained in the present study have shown that the compound, musizin targeted those bacterial enzymes that are crucially involved in cell division, adhesions, biofilm formation and virulence determinants in the selected human bacterial pathogens.

The docking score obtained for the active compound musizin with all the selected target proteins was found to be near to that of the score obtained with gentamycin and streptomycin. The ligand efficiency of musizin has also been found to be significantly high which suggested that the compound could be a potential inhibitor and could be used for the rational drug design for targeting bacterial pathogens. The above-mentioned findings advocate that the isolated active compound musizin might also act by the same mechanism of the standard antibiotics and will reduce the adverse effects.

## Conclusion

A ligand molecule, musizin, isolated and purified from the aerial parts of the plant R. wightii was found to be a potential antibacterial compound which inhibited the growth of both gram-positive and gram-negative bacteria and satisfied the ADMET properties. Docking studies confirmed that thecompound interacted with all the target receptor proteins such as algR (*P. arginosa*), divIVA (*S. fecalis*), icaA (*S. aureus*) and plcR (*B. cereus*) and has higher potential with least binding energy and ligand efficiency compared with the standard antibiotics.Even though the standard antibiotics, streptomycin and gentamycin are commercially available in the market which are not satisfied the ADMET properties. Therefore, we conclude that musizin could be a potential inhibitor to prevent the growth of pathogenic bacteria and serve as an ideal antibiotic in the near future.

## Figures and Tables

**Table 1:** ADMET and physic-chemical properties

Compound Name	Mol. Weight	cLogP	cLogS	H- acceptor	H-donor	Drug likeliness	Mutagenic	Tumorigenic	Reproduction effect	Irritant	Drug Score
Musizin	216.235	2.3777	-3.658	3	2	-1.8529	none	none	none	none	0.4838
Gentamicin	461.473	-1.245	-4.48	12	6	0.70349	High	High	None	High	0.1181
Streptomycin	581.574	-8.208	0.965	19	14	1.9975	none	none	none	High	0.3587

**Table 2:** Molecular docking data

Ligand	Protein (Model)	Binding amino acid Residues	Binding Energy (kcal/mol)	Inhibition Constant uM	VDW_HB desolv_energy (kcal/mol)	RMSD Value (Å)	Ligand efficiency
Musizin	algR	GLU`9/OE1, ARG`15/HE, LYS`102/HZ2	-4.51	494.53	-4.72	59.3	0.28
Gentamicin	″	ARG`15/HE, ASP`54/OD2	-4.37	628.24	-6.03	59.13	0.13
Streptomycin	″	ASP`8/OD1 , GLU`9/OE1 , ARG`56/O, HIS`84/HE2, LYS`102/HZ2	-4.28	728.2	-5.98	69.19	0.11
Musizin	divIVA	PHE`13/O/HN	-6.09	34.27	-6.81	30.17	0.38
Gentamicin	″	PHE`13/O, VAL`25/O	-7.46	3.4	-9.53	22.95	0.23
Streptomycin	″	GLU`12/OE2 , PHE`13/O/HN, GLU`30/OE2	-3.47	2.84	-6.14	30.17	0.09
Musizin	icaA	LYS`189/HZ1, SER`202/O/HG	-5.79	57.28	-6.2	81.55	0.36
Gentamicin	″	LYS`189/HZ1, THR`200/HN, SER`202/O	-6.8	10.32	-8.84	82.36	0.21
Streptomycin	″	ASP`88/OD2, GLU`110/O, ASN`111/O, ASP`220/OD1, GLU`226/OE1/OE2,	-4.42	572.72	-5.52	91.44	0.11
Musizin	plcR	GLU`1D/OE1, LYS`87/HZ2	-5.06	195.35	-5.35	52.46	0.32
Gentamicin	″	LYS`87/O, GLU`271/O	-3.93	1.32	-5.91	63.13	0.12
Streptomycin	″	GLU`193/OE1/OE2, ILE`229/O, ASN`230/O, SER`231/HG	-3.45	86.3	-4.49	60.64	0.14
Musizin	treC	ASN`63/1HD2, HIS`105/HE2, GLN`168/OE1/2HE2	-5.63	74.94	-6.47	52.13	0.35
Gentamicin	″	TYR`65/OH, GLN`168/2HE2, ASP`200/OD2, THR`255/OG1, ASP`325/OD2	-6.04	37.27	-8.07	57.63	0.18
Streptomycin	″	ASP`200/OD2, SER`253/O, ASN`323/1HD2, HIS`324/HE2, ASP`325/OD2, GLN`326/1HE2, ARG`410/1HH1, LYS`281/HZ1	-6.31	23.89	-6.71	54.42	0.16
Musizin	ftsl	ARG`71/HN3, SER`85/HG, ASP`220/OD1, ILE`221/HN	-5.03	204.48	-5.39	78.13	0.31
Gentamicin	″	ARG`71/O/HN1, TYR`214/O, GLY`215/O, ASP`220/OD1, ILE`221/O,	-4.73	309.02	-6.9	82.23	0.15
Streptomycin	″	GLY`205/O, GLU`206/OE2, ARG`207/HN, VAL`209/O	-4.77	72.95	-6.82	95.67	0.2

## Abbreviations

algR: alginate biosynthesis regulatory protein
divIVA: Cell division protein DivIVA
ftsl: cell division protein Ftsl
icaA: Poly-beta-1,6-Nacetyl-D-glucosamine synthase
plcR: Phospholipase C accessory protein
treC: Trehalose-6-phosphate hydrolase
